# Environments for Healthy Aging: Linking Prevention Research and Public Health Practice

**DOI:** 10.5888/pcd10.120244

**Published:** 2013-04-18

**Authors:** Rebecca H. Hunter, Lynda A. Anderson, Basia Belza, Kristin Bodiford, Steven P. Hooker, Chris S. Kochtitzky, David X. Marquez, William A. Satariano

**Affiliations:** Lynda A. Anderson, Centers for Disease Control and Prevention and Emory University, Atlanta, Georgia; Basia Belza, University of Washington, Seattle, Washington; Kristin Bodiford, Community Strengths, Alamo, California; Steven P. Hooker, Arizona State University, Phoenix, Arizona; Chris S. Kochtitzky, Centers for Disease Control and Prevention, Atlanta, Georgia; David X. Marquez, University of Illinois at Chicago, Chicago, Illinois; William A. Satariano, University of California, Berkeley, Berkeley, California.

## Abstract

Safe and well-designed community environments support healthful behaviors that help prevent chronic conditions and unintentional injuries and enable older adults to be active and engaged in community life for as long as possible. We describe the work of the Healthy Aging Research Network (HAN) and partners over the past decade to better understand place-based determinants of health and translate that knowledge to real-world practice, with a focus on environmental strategies. Using key components of the Knowledge to Action framework, we document the importance of a sustained, multidisciplinary, collaborative approach and ongoing interaction between researchers and communities. We share examples of practical tools and strategies designed to engage and support critical sectors with the potential to enhance the health and well-being of older adults and their communities. We conclude with a description of lessons learned in facilitating the translation of prevention research into practice.

## Introduction and the Knowledge to Action Framework

Physical activity, social engagement, and a healthful diet help prevent chronic conditions and increase the longevity and quality of life of older adults (1–3). The importance of physical and social environments on human behavior and health is also well recognized. There are demonstrable cumulative environmental effects on the aging process and the health and functioning of older adults (4). 

The United States will experience in the next 2 decades what has been described from a worldwide perspective as “one of the most daunting challenges of this century” — the unprecedented population growth, from 39 to 70 million, of people aged 65 or older (5). Corresponding growth in the number of older adults with disabling conditions can also be expected; in recent years, more than half of US adults aged 65 or older were reported to have 1 or more disabling conditions (6). Older adults vary in their susceptibility and exposure to unsafe or constraining environments, and those with chronic diseases or functional limitations may be even more adversely affected than their peers by environmental problems (7).

Practices and policies that support safe, age-sensitive, and fully accessible environments help ensure the healthiest possible aging and enable older adults to remain actively engaged in their communities. However, serious concerns exist about our preparedness to meet the challenges of population aging. According to the National Association of Area Agencies on Aging (n4a), in many US communities, “advancements are nowhere near the level of progress that has to be made to ensure that communities are livable for people of all ages” (8). The need to address such challenges underscores the urgency to translate prevention research into action and to define and test effective ways to reach key communities of practice, not only in public health but also in disciplines such as city planning, engineering, and architecture.

The Centers for Disease Control and Prevention’s (CDC’s) Healthy Aging Research Network (HAN) (www.cdc.gov/aging/han/index.htm), which works to better understand place-based determinants of healthy aging and translate findings into practice and policy, is well-positioned for such work. It has member centers from 7 US academic institutions, other university affiliates, and community, state, and national partners working to advance science toward action and policy in support of healthy aging (www.cdc.gov/aging/han/map.htm). HAN conducts research, develops and evaluates initiatives promoting healthy aging, and translates and disseminates science into sustainable, evidence-based public health programs and system-level strategies. HAN focuses on communities and populations that have a disproportionate prevalence of illness (9); consistent with US law, HAN does not use federal funds to directly or indirectly influence federal, state, or local legislation. HAN recognizes the importance of environmental facilitators and barriers to healthful behaviors and community engagement in the healthy aging process (9). Its work, as depicted in a modified Knowledge to Action framework (Figure) (10), reflects a distinct pattern of transition from applied research to translation, with the goal of informing practice and policy. 

**Figure:**
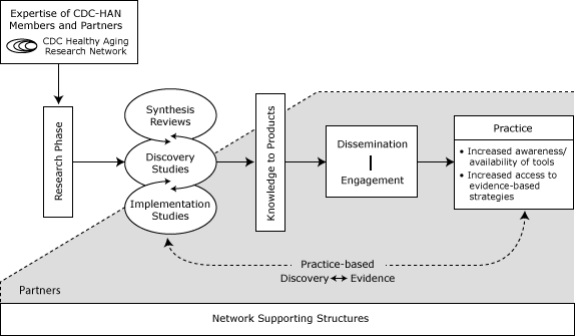
Healthy Aging Research Network Environmental Initiatives: Moving Knowledge to Practice. The HAN Environmental Initiatives Framework is based on the Knowledge to Action Framework (10), highlighting research, knowledge to products, dissemination, partner engagement, and practice effects.

In this article, we use the framework to present the activities and lessons learned from a series of environmental initiatives conducted over 10 years. We briefly describe the network, including our members and partners, and the supporting structures that fund and help sustain our work. We then describe the development of a research agenda and select applied research activities related to synthesis reviews, discovery studies, and implementation studies. We next discuss how research activities led from knowledge to products and describe dissemination and engagement activities. We note the critical role of partners, many of whom are stakeholders with a long-term focus on healthy environments. Finally, we describe the effects of this work on practice, the ongoing influence of practice-based discovery and evidence on further research and dissemination, and the implications for future work.

## Supporting Structures and Partnerships

Being part of an ongoing network has several advantages. First, CDC’s Healthy Aging Program has provided core funding for HAN for more than a decade, allowing for member continuity and development of long-term working relationships. These factors undergird a shared longitudinal vision, support cross-site collaboration, and allow leveraging of resources. Second, network sites are widely distributed across the United States, from urban Seattle to rural South Carolina. These diverse locations represent a range of population groups and community characteristics. Moreover, HAN faculty and established partner organizations, such as the National Council on Aging and the National Association of Chronic Disease Directors, contribute interdisciplinary and cross-sector viewpoints essential for addressing environmental issues. Active community advisory boards anchor HAN sites, and their members provide real-world perspectives and ensure a strong connection to practice. Third, the network has an infrastructure for logistical support and communications through topical workgroups. Taken together, these provide continuity, strengthen capacity, and integrate accomplishments and lessons learned into new initiatives.

Engaging external partners that share an environmental focus (eg, the US Environmental Protection Agency, Easter Seals) is pivotal to moving knowledge into action. Such organizations help frame issues from a national perspective and identify where and how HAN can best contribute. These collaborations result in more influence and expanded reach than are achievable by a sole entity.

## Research Phase

In the research phase of the Knowledge to Action cycle, HAN builds evidence by synthesizing reviews of research findings and conducting discovery and implementation studies. To understand the science of environmental influences and healthy aging, we began more than 10 years ago to examine the literature, focusing on research that investigated environmental determinants of physical activity and looking for knowledge gaps and weaknesses from a public health context. We found a paucity of environmental measures that account for factors relevant for older adults, data pertaining to environmental influences on older adult health, and environmental interventions. These findings led us to conduct research activities related to the environment and older adult physical activity.

In a cross-site discovery study, we surveyed 2,110 community-based organizations in 2002 to determine physical activity program offerings and usage (11). Findings from the 77% of organizations that responded indicated that only 6% of local older adults participated in programs on at least a weekly basis; moreover, some programs such as strength training were not broadly available. Findings made available to study communities immediately influenced practice and program development. A searchable online database tool, Active Options, was subsequently launched to provide access to information about available physical activity programs to older adults and service providers and to facilitate community planning.

Given the demonstrated need for an age- and disability-sensitive built environment measure, we developed the HAN Environmental Audit Tool in 2005 to assess the safety, walkability, and ease of navigation of the built environment for older pedestrians and to ascertain features of the built environment that are important to mobility (12). The Environmental Audit Tool is an adaptation of an existing instrument (13) that was developed on the basis of findings from a discovery study series of older adult interviews and community-based pilot studies across HAN sites. We continue to refine the tool based on new research findings and feedback from other researchers and community users.

The physical activity programs survey and HAN audit tool projects, conducted in geographically diverse HAN sites, showed differences in community environments, particularly between those that were rural and urban. These lessons led us to study types of communities through related initiatives including a review of the effects of the rural built environment on adult physical activity (14), a review on the food environment (15), and audit tool revisions to address rural environment features. We further committed to consider site diversity in subsequent HAN research.

The Robert Wood Johnson Foundation provided funding to 4 HAN sites for a cross-sectional study of 884 people aged 65 or older from diverse communities, assessing how characteristics of the built environment affect older adult patterns of walking and other forms of physical activity. Individual interviews, physical performance measures, and accelerometer data obtained between 2005 and 2007 were complemented by objective environmental measures. Findings indicated the importance of key relationships; for example, living in a residential area, compared with a mixed-use or commercial area, is associated with less time spent walking (16). Older adults with reduced cognitive function were more likely to walk indoors than those with higher functional levels (17), and perceived crime and reduced access to services were associated with higher body mass index (18). Results enhanced understanding of other factors (eg, self-efficacy for walking is linked to reduction or delay of functional limitations [19]) and provided a broader research agenda on mobility.

The National Highway Traffic Safety Administration provided core funding to the University of North Carolina Highway Safety Research Center for Walk Wise, Drive Smart (www.walk-wise.org/), an implementation study conducted in cooperation with the City of Hendersonville, North Carolina. We assessed walking conditions in 10 neighborhoods using the HAN Environmental Audit Tool and resident feedback. These data indicated need for improvement of pedestrian facilities to reduce hazards and barriers to walking by older adults and the need for changes in driver behavior and walking programs tailored to adults with varying fitness levels. The intervention included community education, law enforcement, encouragement of walking, and environmental assessment and modification, including physical improvements to selected routes to improve safety and walkability. The effectiveness of public health professionals, city officials, and informed citizens in making needed environmental changes was a key lesson in the power of cross-sector collaboration.

## Knowledge to Products and Practice Effects

Establishing relationships with communities during our research indicated the need for greater attention to change in environmental practice and policy to broadly affect public health. To move from knowledge to products and practice, we learned to seek out professionals from diverse disciplines who were leaders in environmental and policy change (20) and to engage them in establishing priorities and effective practices. Accordingly, we conducted a research-to-practice symposium, focusing on the challenges of environmental and policy change, the evidence for specific approaches, and promising strategies for practice (5). Conducted in collaboration with the CDC Healthy Communities Program and other sponsors such as AARP and the YMCA, the symposium engaged more than 150 leaders from public health, aging, architecture, business, planning, engineering, recreation, and health care. As a result of the conference, 60% of attendees planned to address their communities’ issues and to initiate programs and practices. Participants identified areas that they would work on, including environmental improvement strategies in their states and communities, and advised us to continue to foster cross-sector collaboration.

Follow-up dissemination activities included presentations to diverse stakeholder groups and a manuscript articulating strategies for improvements in environmentally relevant aging strategies during the second decade of the 21st century (21). With support from the Agency for Healthcare Research, we began an initiative to engage and support additional professionals from diverse disciplines in planning and implementing environmental strategies. In collaboration with Creating Aging Friendly Communities (CAFC) (http://agingfriendly.org/), an Internet community of more than 2,700 participants worldwide, we conducted an online conference series including interactive webinars, on-demand presentations, and selected resources, and facilitated technical support activities. The series brought together researchers, decision makers, and practitioners to advance the implementation of strategies proven to improve public health outcomes. The initiative had excellent reach; 1,206 participants made 4,123 visits to the conference website. Most importantly, participants reported taking steps likely to lead to environmental improvement (55%) and seeing results from those steps (35%). Online resources remain accessible at CAFC.

This interactive dissemination allowed for incorporation of practice-based discovery, yielding fresh insights, increasing understanding of complex practice and policy issues, identifying promising practices, and defining additional areas with potential for impact. As a result, CDC provided support to develop the Environmental and Policy Change Clearinghouse, a searchable, annotated database of online materials about healthy aging and environmental and mobility issues (www.epc-clearinghouse.org.). Recipient of a 2011 APEX Award for Excellence in Publication the clearinghouse includes 130 selected resources and can be readily expanded to include other topics, thereby increasing public access to useful tools and evidence-based strategies. The site had more than 669 visits in its inaugural 3 months.

Follow-up outreach included the public health and aging communities but also extended to key stakeholders (eg, planners, city engineers) via participation in meetings such as New Partners for Smart Growth and ProWalk/ProBike. In these meetings, we highlighted priorities for environmental improvement in areas with potential for high impact, including age- and disability-sensitive neighborhood design and safety, accessible housing stock to support aging in community, fully coordinated mobility and transportation options, and integration of environmental hazard protection into all planning. We also created a series of action briefs (www.prc-han.org/tools-environment#envbriefs) on these issues and broadly disseminated them via partnering organizations. The series, entitled Optimal Living, includes 4 topics: Frameworks to Guide Change, Promising Strategies, Getting Around (focused on mobility), and Home Environments. The briefs focus on promising environmental change strategies for the decade and are intended to prompt action by practitioners and decision-makers in different fields.

Overall, dissemination and engagement efforts have drawn attention to aging-related environmental issues in fields such as urban planning and engineering and in other areas such as falls prevention. For example, the North Carolina Falls Prevention Coalition, in collaboration with public health professionals and city planners, has included neighborhood falls risk assessments into their approach for falls prevention. Other examples are found on the HAN website (www.prc-han.org).

## Knowledge to Action and Back Again

HAN’s environmentally oriented work has been cyclical; each phase in the Knowledge to Action framework informs the next steps. Major concept development initiatives are under way to synthesize knowledge across disciplines in 2 understudied areas: older adult mobility and community wayfinding. Mobility, including both active and passive transportation, and wayfinding, including the system of environmental cues to support mobility, are vital to health and community engagement throughout the lifespan. In support of these initiatives, we are also conducting a study of community wayfinding in Chicago, combining use of the HAN Environmental Audit Tool with older adult interviews and neighborhood wayfinding tasks. 

For more than a decade, HAN has moved science to practice to promote environments for healthy aging. Our experience illustrates the potential of a network of strong member centers and an evolving network of partners with varying missions to achieve common goals. We look forward to continuing to address the challenges of an aging society, helping ensure that our work contributes to the healthiest possible society for all.
